# Acute metabolic effects of tonic‐clonic seizures

**DOI:** 10.1002/epi4.12364

**Published:** 2019-10-22

**Authors:** Robert D. Nass, Berndt Zur, Christian E. Elger, Stefan Holdenrieder, Rainer Surges

**Affiliations:** ^1^ Department of Epileptology University of Bonn Medical Center Bonn Germany; ^2^ Institute for Clinical Chemistry and Clinical Pharmacology University of Bonn Medical Center Bonn Germany; ^3^ Institute for Laboratory Medicine German Heart Centre Munich Munich Germany

**Keywords:** ammonia, biomarker, lactate, seizure, sudden death, SUDEP

## Abstract

**Objective:**

Tonic‐clonic seizures (TCS) lead to metabolic stress and changes in related blood markers. Such markers may indicate harmful conditions but can also help to identify TCS as a cause of transient loss of consciousness. In this study, we hypothesized that the alterations of circulating markers of metabolic stress depend on the clinical features of TCS.

**Methods:**

Ninety‐one adults undergoing video‐EEG monitoring participated in this prospective study. Electrolytes, renal parameters, creatine kinase (CK), prolactin (PRL), lactate, ammonia, glucose, and other parameters were measured at inclusion and different time points after TCS.

**Results:**

A total of 39 TCS were recorded in 32 patients (six generalized onset tonic‐clonic seizures in 6 and 33 focal to bilateral tonic‐clonic seizures in 26 patients). Shortly after TCS, mean lactate, ammonia, and PRL levels were significantly increased 8.7‐fold, 2.6‐fold, and 5.1‐fold, respectively, with levels of more than twofold above the upper limits of the normal (ULN) in 90%, 71%, and 70% of the TCS and returned to baseline levels within 2 hours. Only postictal lactate levels were significantly correlated with the total duration of the tonic‐clonic phase. In contrast, CK elevations above the ULN were found in three TCS (~10%) only with a peak after 48 hours. Immediately after the TCS, hyperphosphatemia occurred in one third of the patients, whereas hypophosphatemia was observed in one third 2 hours later. TCS led to subtle but significant alterations of other electrolytes, creatinine, and uric acid, whereas glucose levels were moderately increased.

**Significance:**

Lactate is a robust metabolic marker of TCS with elevations found in ~90% of cases within 30 minutes after seizure termination, whereas ammonia rises in ~ 70%, similarly to PRL. Phosphate levels show an early increase and a decrease 2 hours after TCS in a third of patients. CK elevations are rare after video‐EEG‐documented TCS, challenging its value as a diagnostic marker.

**Key Points**

- The occurrence and time course of metabolic markers after video‐EEG‐documented TCS were investigated
- Elevations of lactate were found in ~90% of TCS, ammonia and prolactin in ~70%, and CK in ~10%
- Metabolic stress markers, especially lactate, may be useful and complimentary to CK when assessing episodes with transient loss of consciousness due to TCS

## 1 INTRODUCTION

Tonic‐clonic seizures (TCS), that is, generalized onset tonic‐clonic seizures (GTCS) and focal to bilateral tonic‐clonic seizures (FBTCS),^1^ are commonly associated with strong and sustained convulsions of a large number of body muscles along with respiratory arrest and tachycardia, leading to considerable metabolic stress.^2^, ^3^ The resulting changes in respective blood parameters may help in the differential diagnosis on the one hand and in the understanding of the pathophysiology and subsequent potential consequences of TCS including sudden unexpected death in epilepsy (SUDEP) on the other hand.^2^, ^3^

Previous studies often investigated circulating blood markers with the goal to distinguish different causes of episodes of transient loss of consciousness (TLOC). Prolactin (PRL) and creatine kinase (CK) are the most widely used blood markers for purposes of differential diagnosis, with a specificity above 90%, but a lower sensitivity.^3^, ^4^, ^5^ Recent studies suggested that alternative biomarkers may be more accurate to distinguish TCS from other causes of TLOC. For instance, ammonia was shown in several emergency room (ER) studies and one prospective video‐EEG monitoring study to differentiate TCS from other types of TLOC.^3^, ^6^, ^7^, ^8^, ^9^, ^10^, ^11^ Postictal lactic acidosis is a well‐known phenomenon, but few studies have investigated lactate as a diagnostic marker after TCS.^12^, ^13^, ^14^, ^15^ Two previous ER studies suggested that lactate is a reasonably sensitive and specific marker of TCS when TLOC was the chief complaint.^16^, ^17^ The major weaknesses of these studies, however, are that the putative TCS were not unequivocally confirmed by video‐EEG monitoring and that the events following the TCS were not documented (eg, falls and injuries). Furthermore, the actual time course of these biomarkers in the postictal period and its determinant factors are largely unknown.^10^

In the present study, we particularly aimed at the investigation of metabolic consequences and pathophysiological aspects in the aftermaths of TCS. We hypothesized that the occurrence, extent, and time course of abnormally altered metabolic markers depend on the features of prior TCS. Therefore, we have prospectively investigated various blood biomarkers at different time points following documented TCS during video‐EEG monitoring.

## 2 METHODS

Adult patients aged 18 years or older undergoing video‐EEG monitoring in the Department of Epileptology at the University Hospital Bonn for noninvasive presurgical evaluation or syndrome diagnosis and who had a history of previous TCS were asked to participate in this prospective clinical study. The study was approved by the local medical ethics committee (Ethikkommission der Medizinischen Fakultät der Rheinischen Friedrich‐Wilhelms‐Universität Bonn). Only patients who gave informed consent were participated in the study. We did not include patients with a TCS in the week before admission to the video‐EEG monitoring unit.

The standard diagnostic procedure comprised cerebral 3 Tesla magnetic resonance imaging, neuropsychological testing, and continuous video‐EEG monitoring using noninvasive scalp EEG (10‐20 systems with additional temporal electrodes) or, in some cases invasive EEG (hippocampal depth or neocortical subdural electrodes) recordings (Micromed System). The clinical features of TCS (total duration, duration of the tonic‐clonic phase, semiology of TCS) as well as the presence and duration of postictal generalized EEG suppression (PGES) were analyzed blinded to the results of the blood samples.^18^

Blood samples were collected at baseline (when informed consent was given, prior to the occurrence of TCS) and at various time points after the TCS (within 30 minutes after TCS as well as after 2, 6, 24, and 48 hours after a TCS).

We used two serum samples, two EDTA samples, one lithium‐heparin sample, and one sodium‐fluoride sample (S‐Monovette^®^ by Sarstedt^®^). Lithium‐heparin samples for ammonia were cooled at 4°C. Electrolytes were measured using indirect potentiometry. Creatinine, uric acid, blood urea nitrogen (BUN), glucose, lactate, and ammonia were measured by applying VIS photometry. Cystatin C was measured by immunnephelometry. All measurements were conducted on a Dimension Vista® System by Siemens Healthcare Diagnostics^®^. The laboratory measurements were performed at the Institute of Clinical Chemistry at the University Hospital Bonn which is located about 1 km from the Department of Epileptology. Because the mean time from blood sampling to arrival in the laboratory was 64 ± 18 minutes, the sensitive lithium‐heparin samples were cooled immediately after they were drawn. The baseline was drawn when video‐EEG monitoring started after admission 3 ± 2.2 days before the seizure. The first postictal blood sample was drawn 10 ± 8 minutes after the TCS, the second 2 ± 0.2 hours, the third one 6 ± 0.66 hours, the fourth one 23.8 ± 1.56 hours, and final one 46.7 ± 3.7 hours. An overview is provided in Table S1.

Statistics (RM‐ANOVA with Greenhouse‐Geisser correction in case of no sphericity) were performed with IBM^®^ SPSS^®^ statistics, version 24, GraphPad Prism^®^, version 6. For repeated measures ANOVA, *P*‐values <.05 after Bonferroni postprocessing were regarded as significant.

## 3 RESULTS

### 3.1 Patient and seizure characteristics

**Figure 1:**
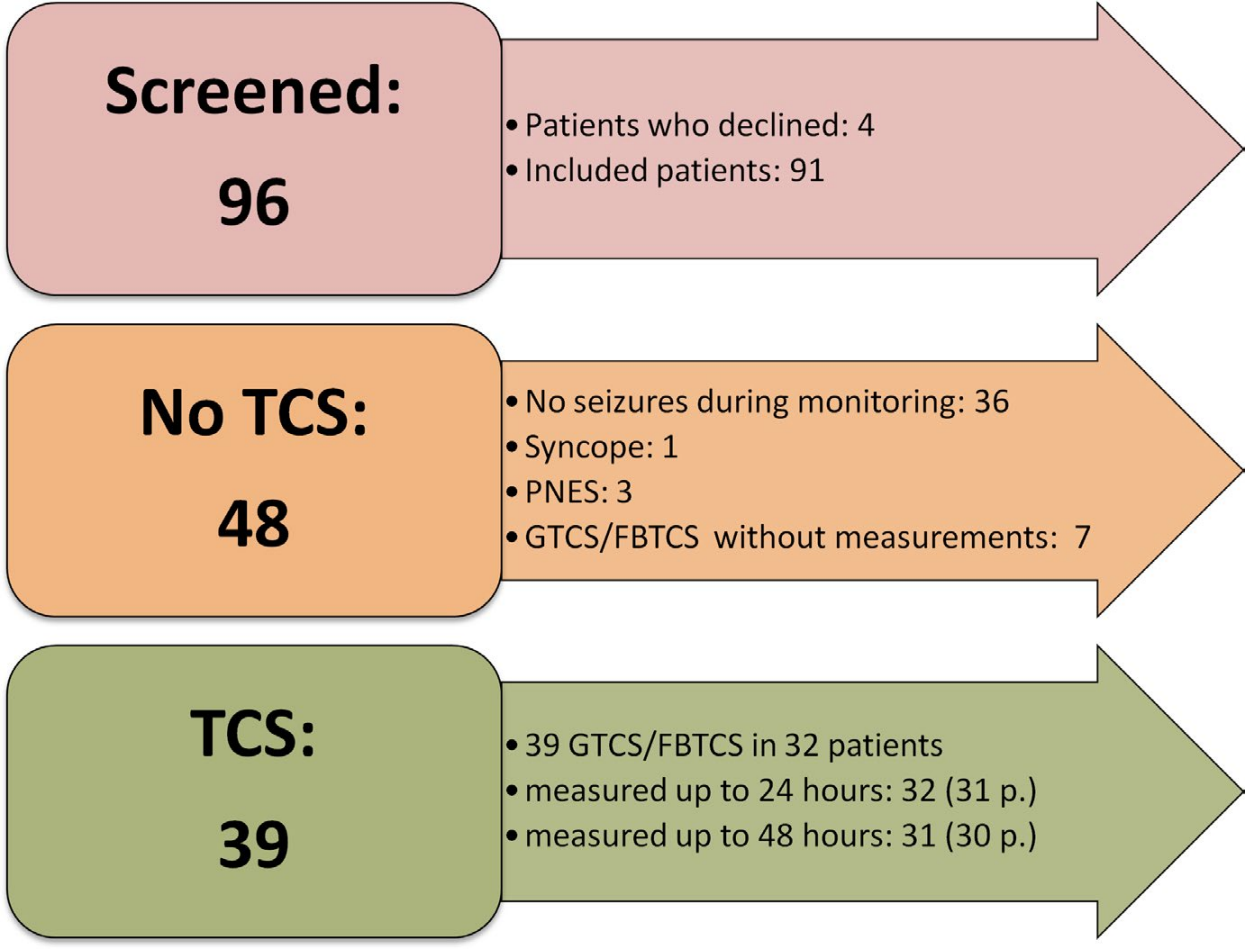
Overview of included patients

Blood samples of a total of 39 TCS in 32 patients were analyzed in this study. These included 6 GTCS from 6 patients and  33 FBTCS from 26 patients. The data on postictal cardiac blood markers in these patients were recently reported.^19^ In 32 TCS from 31 patients, sampling was available up to 24 hours, while the full measurement up to 48 hours was available in 31 TCS from 30 patients. For categorical analyses regarding the frequency of postictal laboratory value changes, we used all 39 TCS, while we used only the 31 TCS from 30 patients that were available up to 48 hours in a repeated measures ANOVA approach. For more details regarding patient inclusion and available blood samples, see Figure 1. Tables 1 and 2 provide an overview of the demographic and clinical characteristics of the patients included in the subsequent analyses. We have also analyzed TCS semiology, but since two of the three TCS types had very small patient numbers, further analysis was not meaningful.^18^

**Table 1:** Overview of demographic and clinical characteristics of the patients who suffered from TCS during video‐EEG monitoring who were included

Demographic parameters	Category	N	Percent
Sex	Female	17	53.1
	Male	15	46.9
Epileptogenic zone	Temporal	17	53.1
	Frontal	4	12.5
	Parietal	1	3.1
	Hemispheric	2	6.3
	Multifocal	2	6.3
	Generalized	6	18.8
Etiology	Unknown	11	34.4
	Genetic/idiopathic generalized	6	18.8
	Hippocampal sclerosis	4	12.5
	Disorders of cortical development	6	18.8
	Benign tumor	2	6.3
	Postinfectious	2	6.3
	Vascular	1	3.1
TCS types	Type I—bilateral symmetric tonic arm extension	4	10.3
	Type II—clonic without tonic extension	2	5.1
	Type III—asymmetric or bilateral tonic arm flexion	33	84.6
Wake state	Asleep	20	51.3
	Awake	19	48.7
Postictal EEG	Alpha	2	5.1
	Diffuse slowing	19	48.7
	PGES	17	43.6
	Undeterminable due to artifacts	1	2.6

Note

The different TCS subtypes were recently defined by Alexandre et al.^17^

Type I: Typical tonic‐clonic seizure with bilateral and symmetric tonic arm extension at the onset of secondary generalization, followed by bilateral and symmetric 4‐limb myoclonic jerk.

Type II: Clonic seizure with bilateral and symmetric 4‐limb myoclonic jerks without tonic arm extension or flexion.

Type III: Tonic‐clonic seizure with asymmetric bilateral tonic arm extension, unilateral tonic arm extension combined with contralateral tonic arm flexion, bilateral tonic arm flexion, or unilateral tonic arm extension, followed by bilateral and symmetric 4‐limb myoclonic jerks and TCS in which the onset was not clear due to blankets covering the camera view, etc.

### 3.2 Creatine kinase (CK) and prolactin (PRL)

**Figure 2:**
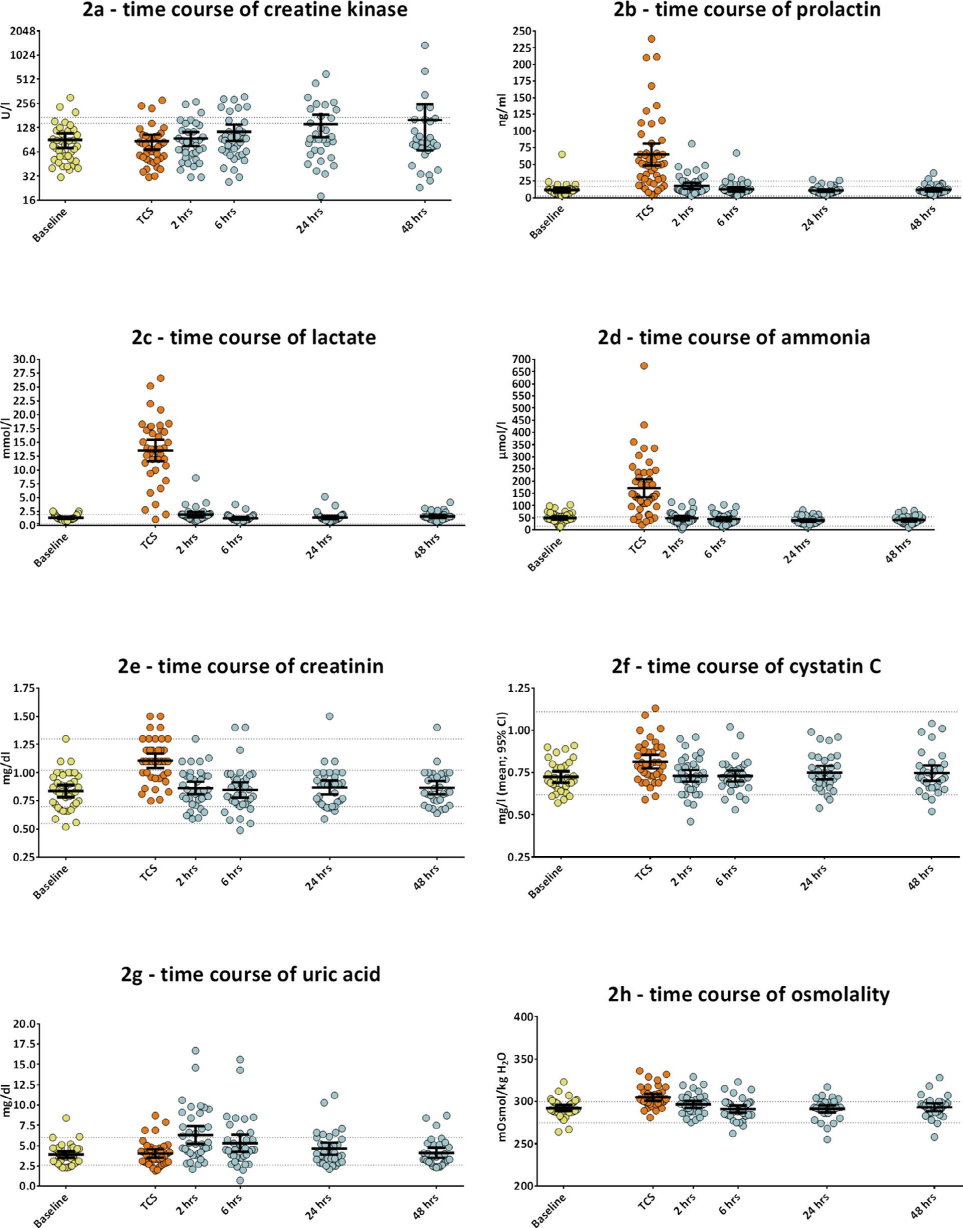
(A‐H) Time course of laboratory values after TCS. All measurements are plotted and used to calculate percentages of elevations, but only complete measurements were used for repeated measures ANOVA. Bars represent means with 95% confidence intervals. The y‐axis in (A) presents the binary logarithm of CK activity in units per liter. All other y‐axes present the concentration of the measured molecules in linear units. Dots present individual measurements. The dotted lines in (A) present the upper limit of normal (ULN) for women (lower line) and men (upper line). The dotted lines in (B) present the ULN for women (upper line) and men (middle line) as well as the lower limit of normal (LLN) for both sexes (lowest line). In (E), the dotted upper lines represent the ULN for men (1.3 mg/dL) and women (1.02 mg/dL), and the lowest dotted line represents the LLN for women and the remaining line represents the LLN for men. In all other figures, the dotted lines present the ULN and LLN for both sexes

At the group level, CK showed a trend to rise after 6 hours with mean CK elevations by 47% 48 hours after the TCS (*P* = .062; Figure 2A). However, only 8.8% of TCS led to CK elevations more than twofold above the upper limit of normal (ULN) after 24‐48 hours, while 45% of the TCS were associated with an increase by more than 5 U/L within 24 hours. PRL rose significantly by fivefold shortly after TCS and returned to baseline levels after 6 hours (*P* < .0001, Figure 2B). About 70% of TCS lead to PRL elevations >2‐fold above the ULN. Levels of CK and PRL levels did not correlate with the TCS duration. For details, see Table S2.

**Table 2:** Continued overview of clinical characteristics of the patients who suffered from TCS during video‐EEG monitoring who were included

	Mean	SD	Median	Minimum	Maximum
Age	33.46	12.13	29.92	18.58	67.30
BMI	25	6	23	18	43
Years since epilepsy diagnosis	17	13	14	0	44
Duration of seizures (s)	156	209	107	25	1155
Duration of tonic‐clonic phase (s)	74	40	69	9	216
Time until reorientation (min)	22.23	19.61	17.12	0.15	96.40

### 3.3 Lactate and ammonia

**Figure 3:**
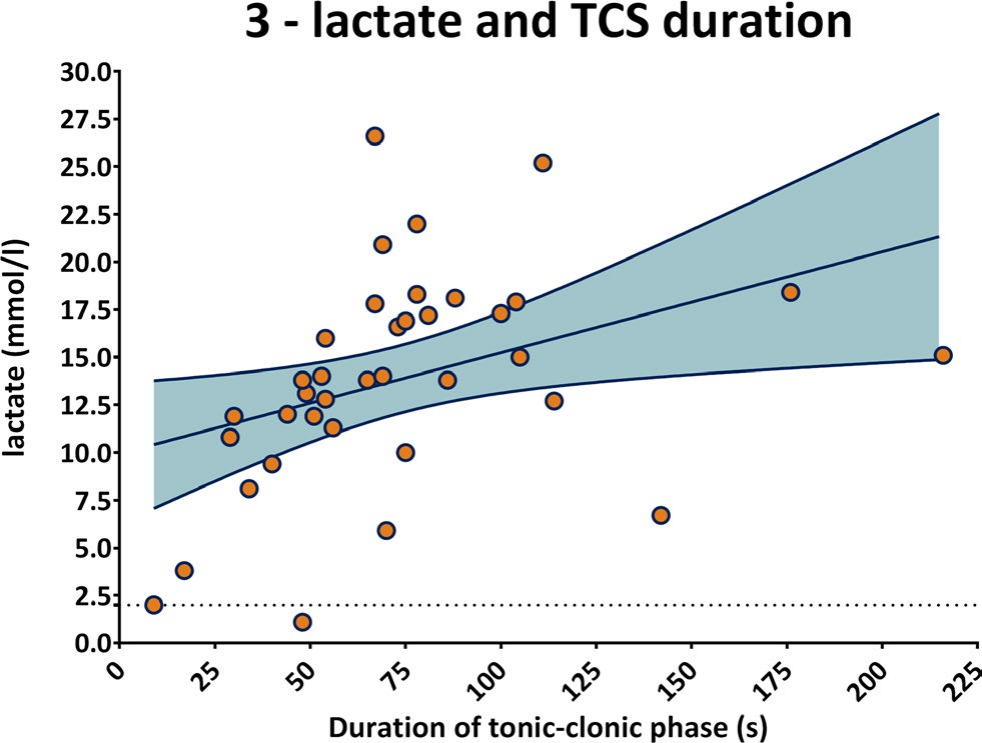
Postictal lactate levels (y‐axis in mmol/l) correlated with TCS duration (x‐axis in s; *r* = .36; *P* = .026). The dotted line at the bottom indicates the ULN of lactate. The gray area represents the 95% confidence intervals of the best fit line

Lactate dramatically rose by 8.7‐fold immediately after TCS and decreased toward baseline levels after 2 hours (*P* < .0001, Figure 2C). Almost 90% of TCS led to lactate elevations more than twofold above the ULN and 94.7% above 2.5 mmol/L, a cutoff value suggested by previous studies.^16^, ^17^ Postictal lactate levels were positively correlated with the duration of the tonic‐clonic phase of prior TCS (*r* = .36; *P* = .026, Figure 3). Ammonia rose significantly by 2.6‐fold immediately after a TCS and returned to baseline after 2 hours (*P* < .001, Figure 2D). More than 70% of TCS lead to ammonia elevations by more than twofold above the ULN. A trend toward higher ammonia levels with longer seizures did not reach statistical significance (*r* = .3; *P* = .07). For details, see Table S2. Since high blood ammonia has detrimental effects on alertness and is associated with postexercise fatigue, we also analyzed the postictal EEG pattern to determine whether higher ammonia levels were associated with PGES.^20^, ^21^ Neither occurrence nor duration of PGES (*P* = .57) nor the time until the patients mental status recovered correlated with ammonia levels (*P* = .54).

### 3.4 Electrolytes

Mean calcium, magnesium, and sodium levels were slightly increased in the immediate postictal period and returned to baseline levels within 2 hours. Phosphate showed the most pronounced changes in all electrolytes. It increased from 1.2 ± 0.25 mmol/L at baseline to 1.44 ± 0.26 mmol/L immediately after the TCS, which lead to mild hyperphosphatemia in one third of patients. After 2 hours, phosphate levels fell to 0.97 ± 0.3 mmol/L, which fulfilled the criteria of mild hypophosphatemia in another third. After 6 hours and at later time points, phosphate levels returned to baseline. There were no significant alterations in potassium and chloride levels (Figures S1E and S1F). Interestingly, mild hyperchloremia was present in 44% of patients throughout the study, and not only immediately after a TCS, as would be expected due to compensatory chloride ion increases as a response to bicarbonate losses during lactic acidosis.^13^ For details, see supporting information online (Figure S1 and Table S2).

### 3.5 Glucose, uric acid, and renal retention values

Mean glucose levels were slightly increased by 14% shortly after TCS, mostly within the normal or mildly elevated range. The renal retention markers creatinine and cystatin c showed a transient but significant rise immediately after TCS (*P* < .001, Figure 2E‐F). Creatinine rose by 32% from 0.83 ± 0.19 mg/dL to 1.1 ± 0.19 mg/dL and cystatin c by 13.3% from 0.72 ± 0.09 mg/L to 0.81 ± 0.12 mg/L (*P* < .0001). Most creatinine values remained in the normal range, but 8 (21.1%) patients fulfilled the criteria for "risk of renal injury" and 1 (2.6%) patient for "acute kidney injury," as described by the acute kidney injury network criteria (AKIN).^22^ Renal retention parameters, however, decreased to baseline levels within 2 hours in all cases. The levels of cystatin c fluctuated mostly within the normal range and blood urea nitrogen remained stable at all times, indicating full compensation of any renal stress.

Uric acid markedly peaked 2 hours after TCS by 61.1% (*P* < .0001, Figure 2G) and returned to baseline levels after 24 hours. About 27% of the patients developed a transient hyperuricemia, but only in 5.4% of the patients hyperuricemia was above twofold of the ULN, which is considered critical and can cause complications such as acute renal failure, which we did not encounter. Osmolality was elevated by 4.3% immediately after TCS (304.4 ± 12.5 mosmol) and returned to baseline levels within two hours (*P* < .0001, Figure 2H).

## 4 DISCUSSION

The major findings of the present study are that profound lactate elevations occur with most TCS and that these alterations are positively correlated with the total duration of TCS. Furthermore, in contrast to most previous reports, CK elevations are rare after documented TCS, challenging its value as a diagnostic marker of TCS.

### 4.1 Study limitations

Investigations of laboratory markers are subject to inherent limitations, for example, due to circumstances of blood collection, transport, and storage of the samples. For instance, the ammonia levels showed a trend to correlate with the transport time to the laboratory (*r* = 0.3; *P* = .07) and elevated ammonia levels above the ULN (but below twice the ULN) were found in about 30% of the patients at baseline or more than 24 hours after the seizures. The blood samples were taken during daily clinical practice, so that the time of sample collection and the seizure was variable to some extent. However, analysis of variable collection and transport times did not reveal major effects on any blood marker apart of the trends in ammonia levels, strengthening the validity and conclusions of our study. Importantly, our study was not designed to assess the actual value of the investigated blood markers in the differential diagnosis of TLOC. Hence, we did not include other patient groups with psychogenic nonepileptic events, syncope, or physiological exercise.

### 4.2 Tonic‐clonic seizures‐related metabolic stress

Tonic‐clonic seizures dramatically increase the energy demands of the brain and skeletal muscle. The changes in metabolites related to TCS show striking similarities to those found in extreme physical exertion, such as in athletic sprinting. A very important difference, however, is that an athlete has full mental control over voluntary movements allowing adjustments of physical activity to warning signals such as fatigue, pain, or shortness of breath, while the total loss of control during a TCS may lead to injuries such as vertebral fractures^23^. During a seizure, the brain will use additional energy in a situation of apnea, whereas an athlete can adjust his breathing patterns to the metabolic needs. About 70% of the TCS in our study were accompanied by cyanosis and 97% received oxygen supplementation, which is part of our nursing standard after TCS. Finally, muscle contractions during physiological and voluntary activity are different from those during TCS, which may also partially explain, for example, the dramatic increase in lactate and other markers during a rather short time period of muscle activity.^24^

Assuming that the pattern of metabolic stress in TCS is to some extent comparable to that of maximum physical efforts, for example, during a sprint of 100‐800 m, muscular ATP turnover increases 1000‐fold.^25^ During the first seconds, most ATP is provided by the fast, 1‐step reaction phosphagen system (creatine kinase, adenylate kinase, and AMP deaminase reactions), which leads to elevated phosphate and creatinine blood levels. The slower, multiple‐step anaerobic glycolytic reactions take over as the main source of ATP production after 10‐20 seconds. Lactate, the end product of anaerobic glycolysis, is released into the bloodstream. The lactate levels found in our study are as high as or higher than those after sprint races in athletes of various disciplines.^26^, ^27^ Free protons from anaerobic glycolysis cause metabolic acidosis, which drives the AMP deaminase reaction of the purine nucleotide cycle, leading to increased ammonia and uric acid production, which are subject to hepatic metabolization and renal excretion, respectively.^27^ During intense exercise, liver and renal perfusion are reduced, further contributing to rising levels of ammonia and uric acid, as well as higher retention of creatinine and cystatin c that we found.^28^ Unlike physiological exercise, the level of metabolic acidosis in TCS may be aggravated by respiratory acidosis.

Aerobic, mitochondrial respiration of lactate, glucose, and fat becomes a prominent ATP source only a minute or more after the onset of intense exercise. Since most TCS are shorter than 2 minutes, most metabolic needs are met by the phosphagen and anaerobic glycolytic systems. Aerobic energy systems become more important in the postictal phase, in which heavy, deep breathing is common to counter the oxygen debt as well as the combined metabolic and respiratory acidosis. During this phase, circulating lactate is oxidized or used for hepatic gluconeogenesis. Gluconeogenesis is further increased by the high levels of catecholamines released during TCS, which may account for the slight increase in glucose seen in our study.^19^, ^29^

The transient hyperammonemia seen in TCS is similar to or exceeding the one observed in bouts of maximum effort exercise of similar length to a TCS.^26^, ^28^ Apart from skeletal muscle, lactate and ammonia may be released to a lesser extent from the brain itself.^6^, ^7^, ^9^, ^10^, ^11^ Experimental data in rats show that cerebral ammonia and lactate production is increased in seizures.^28^, ^30^ Transient hyperuricemia after TCS is rarely analyzed in clinical practice.^31^ Rising uric acid levels have been observed not only in the muscular exercise^32^, ^33^, ^34^, ^35^, ^36^ but also in the seizing rat brain^30^, ^37^, indicating that both organ systems are potential contributors. High postictal uric acid can lead to acute renal failure independently from rhabdomyolysis.^38^, ^39^ The changes in electrolytes and osmolality are likely to reflect fluid shifts caused by the metabolic changes discussed above.

### 4.3 Prolactin and creatine kinase as laboratory markers of TCS

Transient PRL elevations were found in 70% of TCS, consistent with previous studies.^5^, ^40^, ^41^ In contrast, CK elevations were only detected in ~10% of TCS, which is surprisingly low as compared to results of ER studies reporting relevant CK elevations in 40%‐60% of TCS.^3^, ^4^ However, previous studies in the setting of an epilepsy monitoring unit reported significant CK elevations after 14%‐19% of the TCS, which questions the predictive diagnostic value of CK elevations.^40^, ^41^ It is unknown why CK levels are higher in ER studies than in video‐EEG monitoring studies. Glotzner and Chesson already showed that falls and injuries are no prerequisite to CK elevations and rhabdomyolysis, a finding that we recently replicated in a retrospective ER study.^42^, ^43^, ^44^ One reason could be that ER studies include alcohol‐ or substance‐related TCS and maybe falls and injuries that were not reported or observed. Unprotected lying on hard surfaces instead of a hospital bed may also play a role. Prolonged seizures, seizure series, and status epilepticus are more likely to cause CK elevations than single seizures, and those patients are more likely to be referred to ER. Most of the patients in our study had single seizures only, but the one with the highest CK (1356 U/L) had two TCS within one hour. Finally, the subtype of TCS may be of importance. CK elevations after muscle exercise occur more often with high eccentric as opposed to concentric loads^45^. About 85% of our patients had bilateral or asymmetric tonic arm flexion at the onset of TCS, which may be less eccentric stress on large muscle groups than in other TCS types.^18^

Both laboratory markers display decent specificity, but moderate sensitivity for epileptic seizures. PRL is elevated after epileptic seizures and syncope and quickly returns to baseline levels, which limits its value in the daily clinical practice. Further limitations of PRL are its dependency on the menstrual cycle in women of childbearing age and on circadian rhythm.^3^, ^5^ CK elevations are found hours to days after seizures, but are also increased after muscle damage of other causes, such as alcohol and drug abuse, medications, or myopathies. These possible confounders were not present in any of the studied patients with TCS. CK measurements after TCS have the important additional value to rule out the potentially severe complication of rhabdomyolysis.^3^, ^4^, ^40^

### 4.4 Lactate and ammonia as laboratory markers of TCS

The value of ammonia and lactate in the differential diagnosis of TCS versus other types of TLOC was recently demonstrated in ER studies.^6^, ^7^, ^8^, ^9^, ^10^, ^11^, ^16^, ^17^ In addition to these studies, we provide a detailed time course under video‐EEG‐controlled conditions.

Ammonia was transiently elevated in a similar proportion of patients as PRL shortly after a TCS (~70%). Since ammonia also quickly returns to baseline levels, it does not provide a wider diagnostic time window, which limits its use in the daily clinical practice.^6^, ^7^, ^8^, ^9^, ^10^, ^11^ Elevated levels of ammonia lead to fatigue in athletes and even delirium or coma in hepatic encephalopathy.^28^ In our patient group, however, postictal ammonia levels were not associated with the recovery of mental function or with PGES (as a potential surrogate marker of comatose state). Elevated ammonia levels in patients with epilepsy may be caused by the anticonvulsant drugs valproic acid and phenobarbital^46^. None of the patients with TCS in our study was treated with either of the two drugs, so that this possible confounder is ruled out in our study, but should of course be considered in daily clinical practice.

Serum lactate levels were elevated in ~90% of seizures and returned to baseline within 2 hours, indicating that shorter time windows are needed to use lactate as reliable biomarker of TCS. Orringer and colleagues showed a lactate reduction of 50% within the first postictal hour with a majority of patients still having elevated lactate at one hour.^12^ In previous ER studies, lactate elevations above 2.45 mmol/L for up to two hours were described in 73%‐88% of the patients.^17^ In blood samples taken 2 hours after the TCS, only 16.7% of our patients had lactate levels above 2.5 mmol/L. The advantages of lactate measurements are that they are inexpensive and readily available in point‐of‐care measurements in most ER. In this context, it is noteworthy that a decrease in bicarbonate and an increase in the serum anion gap after TCS were recently described in ER studies, which are likely to reflect shifts of bicarbonate and anions due to seizure‐related lactic acidosis.^47^, ^48^ Postictal intracerebral lactate levels were also shown to be moderately elevated in the cerebrospinal fluid many hours after seizures in up to 28% of those cases who received a lumbar puncture for diagnostic purposes (ie, exclusion of inflammatory CNS processes).^49^

Recently, results of a retrospective ER study suggested hypophosphatemia as a potential marker of TCS in the differential diagnosis of TLOC^50^. Interestingly, we have also found postictal hypophosphatemia in one third of our patients, thus apparently confirming this particular finding. Thanks to our prospective study design, however, we have described the exact time course of phosphatemia and found that in the acute phase (ie, within the first 30 minutes), one third of the patients display an increase in phosphate levels, whereas it only fell below normal levels after 2 hours. This finding underscores the importance of prospective studies under controlled conditions in the process of identification and confirmation of possible biomarkers for seizures.

### 4.5 Potential implications in the context of SUDEP

The most likely pathways leading to SUDEP are postictal respiratory depression, postictal arrhythmias, or a combination of both.^2^ The alterations of electrolytes that we encountered in our study, if further exaggerated, may contribute to alterations in cardiac excitability, which may result in arrhythmias. Postictal hyperkalemia was found in animal models and other studies but was not confirmed in our patient group. Severe lactic acidosis as well as electrolyte changes may affect cardiac function and facilitate onset of cardiac arrhythmias.^51^ We have recently reported cardiac symptoms and cardiac blood markers occurring in the aftermaths of TCS in the same patients.^19^ None of the patients displayed cardiac symptoms during or after TCS. High‐sensitive troponin T indicating cardiac injury was, however, detected in about 25% of the patients following TCS. The occurrence of troponin T was only correlated with elevated levels of dopamine, but not with seizure duration or with lactate levels. Interestingly, metabolic acidosis can increase the respiratory drive, possibly counteracting postictal apnea (which is thought to be the primary driver of SUDEP) to some extent. In summary, the implications of our results for the pathophysiology of SUDEP are limited, as none of the TCS was fatal or associated with life‐threatening cardiorespiratory conditions.

## CONFLICT OF INTERESTS

R. Surges has received speaker fees or honorary for serving on the advisory board from Bial, Desitin, Eisai, LivaNova, Novartis, and UCB Pharma. CE Elger has received speaker fees from Eisai, Novartis, and UCB Pharma. RD Nass has received speaker fees from Eisai. The authors declare that there is no conflict of interest regarding the publication of this paper. We confirm that we have read the Journal's position on issues involved in ethical publication and affirm that this report is consistent with those guidelines.
