# Supplementary figures and images for: Acute metabolic effects of tonic‐clonic seizures
Source: Epilepsia Open. 2019 Oct 22;4(4):599–608. doi: 10.1002/epi4.12364 (PMC6885665; doi:10.1002/epi4.12364)

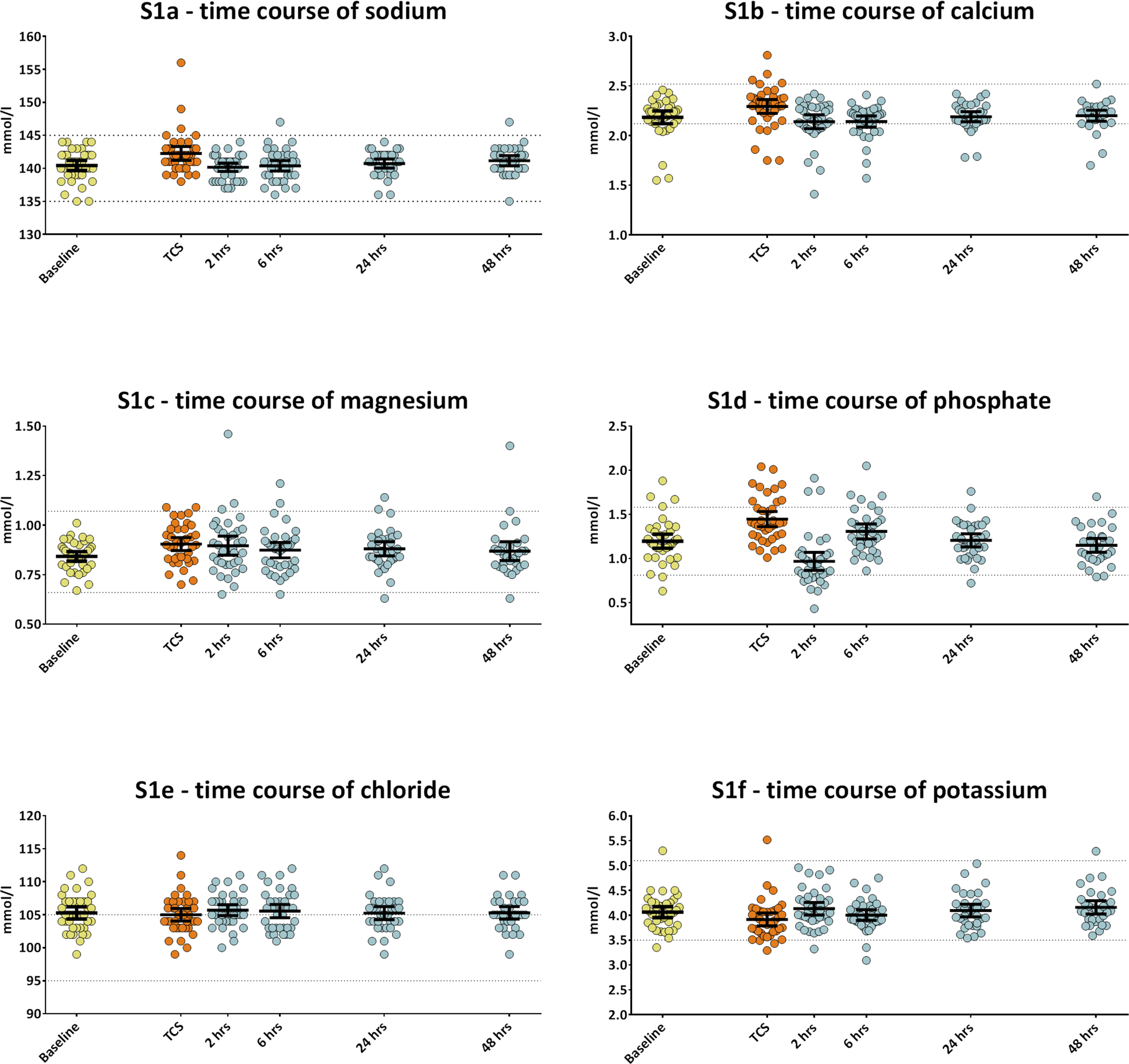

Supplement: Supplementary file 1 [file EPI4-4-0-s001.tif]
