# Supplementary material for: Acute metabolic effects of tonic‐clonic seizures
Source: Epilepsia Open. 2019 Oct 22;4(4):599–608. doi: 10.1002/epi4.12364 (PMC6885665; doi:10.1002/epi4.12364)
Supplement: Supplementary file 2 [file EPI4-4-0-s002.docx]

| Time | Mean | SD | Median | Min | Max |
| --- | --- | --- | --- | --- | --- |
| Transport time from ward to laboratory (min) | 64 | 18 | 64 | 35 | 112 |
| Time from baseline to TCS blood sample (days) | 2.98 | 2.21 | 2.3 | 0.8 | 7.46 |
| time from TCS to first sample (min) | 10 | 8 | 7 | 1 | 35 |
| time from TCS to 2 h sample (hrs) | 2.08 | 0.22 | 2.08 | 1.27 | 2.37 |
| time from TCS to 6 h sample (hrs) | 6.03 | 0.66 | 6.05 | 4.22 | 7.10 |
| time from TCS to 24 h sample (hrs) | 23.82 | 1.56 | 24.06 | 21.10 | 27.98 |
| time from TCS to 48 h sample (hrs) | 46.8 | 3.68 | 47.75 | 37.20 | 49.42 |

*Table S1) Timing of blood samples used in the study.*

|  | Baseline mean (95%-CI) | Early after TCS mean (95%-CI) | 2 hrs mean  (95%-CI) | 6 hrs mean  (95%-CI) | 24 hrs mean  (95%-CI) | 48 hrs mean  (95%-CI) | p-value |
| --- | --- | --- | --- | --- | --- | --- | --- |
| Sodium  mmol/l | 140.44  (139.67-141.2**)** | 142.26  (141.22-143.3**)** | 140.16  (139.53-140.79**)** | 140.39  (139.6-141.18**)** | 140.73  (140.05-141.4**)** | 141.16  (140.38-141.94**)** | **0.02** |
| Potassium  mmol/l | 4.06  (3.95-4.17**)** | 3.92  (3.78-4.05**)** | 4.13  (4.01-4.26**)** | 4  (3.9-4.11**)** | 4.09  (3.97-4.22**)** | 4.16  (4.03-4.29**)** | 0.013 |
| Calcium  mmol/l | 2.18  (2.12-2.25**)** | 2.29  (2.22-2.36**)** | 2.14  (2.07-2.21**)** | 2.14  (2.08-2.2**)** | 2.19  (2.14-2.24**)** | 2.2  (2.15-2.26**)** | **<0.0001** |
| Chloride  mmol/l | 105.28  (104.36-106.2**)** | 105  (104.04-105.96**)** | 105.7  (104.88-106.52**)** | 105.56  (104.55-106.56**)** | 105.24  (104.26-106.23**)** | 105.29  (104.35-106.23**)** | 0.86 |
| Phosphate  mmol/l | 1.2  (1.12-1.27**)** | 1.45  (1.36-1.53**)** | 0.97  (0.86-1.07**)** | 1.31  (1.22-1.39**)** | 1.2  (1.13-1.28**)** | 1.15  (1.07-1.23**)** | **<0.0001** |
| Magnesium  mmol/l | 0.84  (0.82-0.87**)** | 0.9  (0.87-0.94**)** | 0.9  (0.85-0.94**)** | 0.87  (0.83-0.91**)** | 0.88  (0.85-0.92**)** | 0.87  (0.82-0.92**)** | **0.006** |
| Osmolality  mOsm | 292.23  (288.74-295.72**)** | 304.31  (300.21-308.40**)** | 296.51  (292.44-300.58**)** | 291.14  (286.75-295.54**)** | 291.18  (286.91-295.45**)** | 293.16  (288.48-297.85**)** | **p<0.0001** |

Table S2a) Time course of electrolyte measurements in mmol/l, measured after 39 TCSs in 32 patients. Significant p-values (repeated measures ANOVA) are marked in bold.

|  | Baseline mean (95%-CI) | Early after TCS mean (95%-CI) | 2 hrs mean  (95%-CI) | 6 hrs mean  (95%-CI) | 24 hrs mean  (95%-CI) | 48 hrs mean  (95%-CI) | **p-value** |
| --- | --- | --- | --- | --- | --- | --- | --- |
| Creatinin  mg/dl | 0.84  (0.78-0.89) | 1.11  (1.04-1.17) | 0.86  (0.81-0.92) | 0.85  (0.78-0.91) | 0.87  (0.81-0.93) | 0.87  (0.81-0.93) | **p<0.001** |
| BUN  mg/dl | 25.82  (23.42-28.22) | 25.39  (23.20-27.59) | 24.65  (22.31-26.98) | 25.06  (22.46-27.65) | 25.85  (23.20-28.50) | 25.32  (22.28-28.36) | **p<0.0001** |
| Cystatin C  mg/l | 0.72  (0.69-0.76**)** | 0.81  (0.77-0.86**)** | 0.73  (0.70-0.77**)** | 0.73  (0.70-0.76**)** | 0.75  (0.71-0.79**)** | 0.75  (0.70-0.79**)** | **p<0.001** |
| uric acid  mg/l | 3.91  (3.51-4.31**)** | 4.03  (3.52-4.54**)** | 6.32  (5.22-7.41**)** | 5.30  (4.24-6.36**)** | 4.64  (3.89-5.38**)** | 4.12  (3.49-4.75**)** | **p<0.0001** |
| ammonia  nmol/l | 48.26  (41.51-55.00**)** | 173.53  (131.46-215.60**)** | 44.69  (36.78-52.61**)** | 44.03  (36.16-51.90**)** | 38.06  (32.09-44.04**)** | 39.23  (33.17-45.28**)** | **p<0.001** |
| CK  U/l | 88.90  (70.04-107.75**)** | 86.63  (68.34-104.93**)** | 94.05  (75.26-112.84**)** | 113.69  (87.44-139.95**)** | 141.76  (97.33-186.19**)** | 159.03  (66.65-251.41**)** | 0.062 |
| Glucose  mg/dl | 103.68  (90.03-117.34**)** | 116.26  (105.56-126.96**)** | 101.00  (89.54-112.46**)** | 100.58  (90.82-110.33**)** | 96.67  (88.97-104.37**)** | 95.87  (86.88-104.87**)** | **0.001** |
| Lactate  mmol/l | 1.39  (1.22-1.56) | 13.55  (11.62-15.49**)** | 1.97  (1.51-2.44**)** | 1.30  (1.06-1.54**)** | 1.45  (1.13-1.77**)** | 1.65  (1.36-1.95**)** | **p<0.0001** |
| Prolactin  ng/ml | 11.60  (7.95-15.25**)** | 66.89  (49.94-83.84**)** | 24.64  (12.96-36.31**)** | 13.33  (9.28-17.39**)** | 11.44  (9.47-13.42) | 12.99  (10.22-15.76**)** | **<0.0001** |

Table S2a) Time course of other metabolites, measured after 39 TCS in 32 patients. Significant p-values (repeated measures ANOVA) are marked in bold.
