# Supplementary material for: Acute metabolic effects of tonic‐clonic seizures
Source: Epilepsia Open. 2019 Oct 22;4(4):599–608. doi: 10.1002/epi4.12364 (PMC6885665; doi:10.1002/epi4.12364)
Supplement: Supplementary file 3 [file EPI4-4-0-s003.docx]

Supporting information figures S1 a-f) Time course of electrolyte measurements after TCS. The y-axis presents the electrolyte concentration in mmol per liter. Dots present individual measurements. The dotted lines present the upper and lower limit of normal. Bars represent means with 95 % confidence intervals.

Mild but significant increases in electrolyte levels were detected for sodium (S1a; p=0.02), calcium (S1b; p<0.0001) magnesium (S1c; p=0.006). Phosphate showed significant (S1d; p<0.0001) fluctuations in the periictal period ranging from mild hyperphosphatemia postictally to mild hypophosphatemia 2 hours thereafter. Chloride (S1e) and potassium (S1f) remained stable and showed no significant alterations. Of note, mild hyperchloremia was present in multiple patients throughout the study. All measurements are plotted and used to calculate percentages of elevations but only complete measurements were used for repeated measures ANOVA (n=30).
